# Visuo-motor interference is modulated by task interactivity: A kinematic study

**DOI:** 10.3758/s13423-023-02297-z

**Published:** 2023-05-01

**Authors:** Matilde Rocca, Lucia Maria Sacheli, Luca Romeo, Andrea Cavallo

**Affiliations:** 1 Department of Psychology and Berlin School of Mind and Brain, Humboldt University of Berlin, Berlin, Germany; 2 C’MoN, Cognition, Motion and Neuroscience Unit, Fondazione Istituto Italiano di Tecnologia, Genova, Italy; 3 Department of Psychology and Milan Center for Neuroscience (NeuroMi), University of Milano-Bicocca, Milano, Italy; 4 Department of Economics and Law, University of Macerata, Macerata, Italy; 5 Computational Statistics and Machine Learning Laboratory, Center for Human Technologies, Fondazione Istituto Italiano di Tecnologia, Genova, Italy; 6 Move‘N’Brains Lab, Department of Psychology, University of Torino, Torino, Italy

**Keywords:** Motor cognition, Visuo-motor interference, Social interaction, Movement kinematics, Dyadic motor plan, Machine learning, Joint action

## Abstract

Extensive evidence shows that action observation can influence action execution, a phenomenon often referred to as visuo-motor interference. Little is known about whether this effect can be modulated by the type of interaction agents are involved in, as different studies show conflicting results. In the present study, we aimed at shedding light on this question by recording and analyzing the kinematic unfolding of reach-to-grasp movements performed in interactive and noninteractive settings. Using a machine learning approach, we investigated whether the extent of visuo-motor interference would be enhanced or reduced in two different joint action settings compared with a noninteractive one. Our results reveal that the detrimental effect of visuo-motor interference is reduced when the action performed by the partner is relevant to achieve a common goal, regardless of whether this goal requires to produce a concrete sensory outcome in the environment (joint outcome condition) or only a joint movement configuration (joint movement condition). These findings support the idea that during joint actions we form dyadic motor plans, in which both our own and our partner’s actions are represented in predictive terms and in light of the common goal to be achieved. The formation of a dyadic motor plan might allow agents to shift from the automatic simulation of an observed action to the active prediction of the consequences of a partner’s action. Overall, our results demonstrate the unavoidable impact of others’ action on our motor behavior in social contexts, and how strongly this effect can be modulated by task interactivity.

**Supplementary Information:**

The online version contains supplementary material available at 10.3758/s13423-023-02297-z.

Observing other people’s movements is an active process that involves, besides visual regions, the same cerebral motor regions that we would use to perform the observed movement (Rizzolatti & Sinigaglia, 2016). This process appears to be beyond our direct control and has noticeable effects on our overt motor behavior. Indeed, when we act during or in response to another person’s movement, the observed action has an impact on the execution of our own action (Brass et al., 2000, 2001; Craighero et al., 2002), a phenomenon often referred to as *automatic imitation* (Cracco et al., 2018), or *visuo-motor interference* (Blakemore & Frith, 2005; Kilner et al., 2003). Converging evidence indicates that, as a result of visuo-motor interference, action execution is facilitated by the observation of *congruent* actions and disrupted by the observation of *incongruent* actions. Incongruency effects have often been observed as slower reaction times, enhanced motor variability and reduced precision (Brass et al., 2001; Kilner et al., 2003). Some studies have linked the phenomenon of visuo-motor interference to a motor contagion effect, by which the observed movement is incorporated into motor execution. This effect has been observed in the kinematic unfolding of the performed movement, which can show a pattern of kinematic similarity to the observed one (Dijkerman & Smit, 2007; Forbes & Hamilton, 2017; Griffiths & Tipper, 2009).

Recently, a new question has been raised, led by a growing interest in understanding human cognition from a *social* perspective (Kingsbury & Hong, 2020; Schilbach et al., 2013): Is the extent of visuo-motor interference modulated when we actively *interact* with others? This question is critical for understanding which cognitive mechanisms allow us to interact effectively with others. Humans possess early-developed, specie-unique prosocial tendencies and sharing abilities (Tomasello et al., 2005; Ulber & Tomasello, 2020), which enable them to infer other’s intentions and future actions while cooperating, as recently suggested by theoretical and computational models (Maisto et al., 2022; Pesquita et al., 2018; Wu et al., 2021). However, the role played by the motor system in this context still remains an open question.

To address this issue, recent studies have compared the effects of action observation on behavior in interactive and noninteractive settings using the scenario of *joint actions* as a model for cooperative interactivity (Sebanz et al., 2006; Sebanz & Knoblich, 2021). Joint actions are test-case situations, in which two or more individuals coordinate their actions to produce together a change in the environment. Although the model of joint action cannot account for the entire range of complex interactions that are possible between individuals (e.g., linguistic conversation, competition), they have been proved to be a good model to understand the fundamental mechanisms that arise during cooperative social interactions (Obhi & Sebanz, 2011). Compared with a noninteractive context—where two agents pursue individualistic goals—during a joint action two agents *share* the same goal, which can only be achieved by the two coordinated actions of the pair. Therefore, the action of the other is necessary to achieve the desired *shared* outcome, and it thus needs to be taken into account and monitored (Vesper et al., 2010, 2017).

In this regard, some studies show that, compared with a noninteractive scenario, visuo-motor interference is reduced during joint actions. They suggest that, during joint actions, we shift from the automatic simulation of an observed action to the active prediction of the consequences of a partner’s action. In this framework, the other’s action is not disruptive, as it becomes part of a dyadic motor plan in which it is processed in terms of its predicted effects on the environment (Clarke et al., 2019; Sacheli, Arcangeli, & Paulesu, 2018a; Sacheli, Verga, et al., 2019b).

However, other studies show that, compared with a noninteractive setting, a joint action setting enhances the effect of visuo-motor interference, because the action performed by the other becomes part of our own action goal: it thus needs to be represented and monitored, leading to a higher motor activation during action observation and thus to greater visuo-motor interference (della Gatta et al., 2017; Era et al., 2020).

This controversial evidence might be explained by the different way the construct of joint action has been operationalized. Whereas the studies that found high visuo-motor interference considered joint actions in which a feature of the other’s *movement* (e.g., trajectory) was necessary to achieve the common goal (e.g., perform Movement A, while the other performs Movement B, to achieve AB), the studies that found low visuo-motor interference considered joint actions in which the *outcome produced by the other’s movement* (e.g., musical note) was the key to achieve the common goal (e.g., perform Movement A to produce X, while the other performs Movement B to produce Y, to achieve XY).

With this in mind, in the present study, we aimed to shed light on the potential modulations of visuo-motor interference during joint actions by assessing its presence during three different interactive scenarios, in which the action of the other was either (i) irrelevant to the agent's *individualistic goal* (noninteractive condition), (ii) necessary to achieve a *movement configuration* together (joint-movement condition), or (iii) necessary to produce a *joint outcome* together, resulting from the movement configuration achieved (joint-outcome condition).

To this end, we used a motion-capture system to record the kinematics of sequential reach-to-grasp movements performed by pairs of agents (i.e., a participant and a confederate). First, the confederate reached for and grasped an object with a precision grip (PG) or with a whole-hand prehension (WHP). Then, the participant performed the same (congruent) or opposite (incongruent) action on a second object (Fig. 1).

**Fig. 1:**
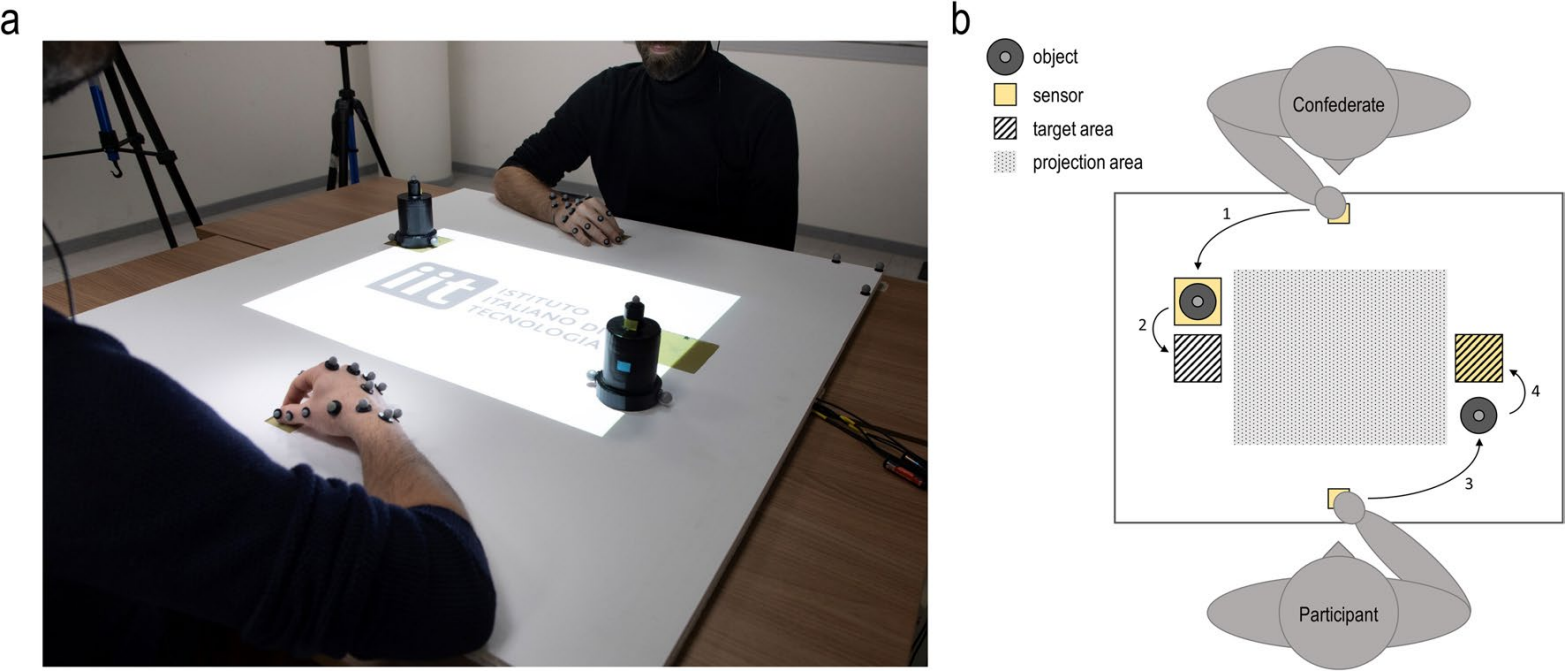
Experimental setup. Panel (**a**) shows a photo of the experimental setup, in which the participant and the confederate keep their hands in their respective starting positions. In front of the two agents are placed the two objects used during the experiment. The objects were designed to be grasped with either a precision grip or a whole hand prehension. The bright area represents the projection area. During the experiment, the projection area was dark. Projections were used only during catch-trials, during error trials, and during the *joint outcome* condition. Panel (**b**) shows a schematic (not in scale) representation of the experimental setup. The numbers refer to the order in which the actions were performed during each motor sequence (i.e., trial). Press and release sensible sensors were placed in strategic positions to control for correct performance during each trial

Given the complexity and the intrinsic multidimensionality of movement kinematics, we used a machine learning approach to quantify at single trial level the presence of visuo-motor interference in the participants’ reach-to-grasp movements across the different interactive scenarios. Compared with previous studies, this novel approach allowed us to assess the presence of visuo-motor interference in the actual kinematic unfolding of the participants’ movements. While we expected high visuo-motor interference in the *noninteractive* condition (Dijkerman & Smit, 2007; Kilner et al., 2003), the possible reduction or enhancement of visuo-motor interference in the *joint-movement* and *joint-outcome* conditions would clarify whether its underlying mechanisms are also and primarily recruited during interpersonal coordination (della Gatta et al., 2017), or not (Clarke et al., 2019; Sacheli, Arcangeli, & Paulesu, 2018a), being interpersonal coordination based on qualitatively different motor processes. The primary aim of our study was to address this question by analyzing participants’ kinematics through a machine learning approach.

A secondary aim of our investigation, irrespective of a difference between conditions, was to explore, in the movements performed by the participants, the specificity and temporal evolution of the visuo-motor interference effect. This interest was led by the evidence suggesting the presence of kinematic similarity between actor and observer, as a result of visuo-motor interference: Indeed, the nature of our data allowed us to focus on fine grained aspects of movement unfolding, which were recorded from both the participants and the confederate. In particular, we explored whether visuo-motor interference was driven by the specific kinematic properties of the counterpart’s movement, as suggested by recent studies (Dijkerman & Smit, 2007; Forbes & Hamilton, 2017; Griffiths & Tipper, 2009; Hardwick & Edwards, 2011), and if so, if this effect increased over time (Rocca & Cavallo, 2021).

## Methods

### Participants

Sixteen participants, each performing 240 experimental trials (*N* trials = 3,840), took part in the experiment (nine females; ages 25–40 years; mean age = 29.06 years; *SD* = 4.34). The sample size was determined based on previous studies that used trial-wise analyses on movement kinematics data (Ansuini et al., 2015; Cavallo et al., 2016). The dataset was collected before any analysis began, and no data was added after the beginning of the analyses. All participants were right-handed, had normal or corrected-to-normal vision, and no history of neurological disorders. The study was approved by the local ethics committee (ASL3 Genovese). All participants provided written informed consent in accordance with the principles of the revised Helsinki Declaration (World Medical Association, 2013) and were naïve with respect to the purpose of the experiment.

### Procedure

The experiment was explained to participants as a simple sequential task. Participants were told that the general rule of the task was to reach and grasp—either with a PG or with a WHP (depending on the experimental trial)—the object that was in front of them, and to place it on the target area as quickly and accurately as possible. Participants could start their action only after having observed the confederate reaching and lifting his own object.

At the beginning of the trial, both the confederate and the participants were instructed to hold the same starting position, which consisted in keeping the left hand on the left knee, the right arm oriented in the parasagittal plane passing through the shoulder, the forearm pronated, the wrist resting on the table, and the hand in a semipronated position, with the thumb and the index finger opposed and pressing the small sensor positioned in front of them (Fig. 1a). Only when the two agents were in their starting positions, they heard on their headphones the instructions relative to the type of grasp to perform during the sequence.

These instructions were given to both the participant and the confederate in the form of colors (e.g., “blue” = grasp the object with a precision grip). The two types of grasp (i.e., PG or WHP) were in fact associated with two colored labels (i.e., blue and yellow; 1.5 cm × 1 cm) placed on each of the two objects. One label was applied on the upper (i.e., small) part of the object, and the other on the bottom (i.e., large) part. The association between color and upper/lower part of the object was counterbalanced between participants. This association was made in order to equate the instructions given to the participants in all the experimental conditions, and to elicit a difference in perceived interactivity between conditions (please see below the difference between noninteractive, joint-movement, and joint-outcome conditions).

The confederate heard only one color, such as the one associated to the type of grasp he had to perform. Participants heard instead two colors: The first color described the type of grasp that the confederate had to perform, and the second color described the type of grasp that the participant had to perform. After the instructions (jittered interval: 1000, 2000 or 3000 ms), the confederate heard a beeping sound (frequency: 750 Hz; duration: 150 ms). This sound was his GO signal: When he heard it, he could release the sensor and start to reach, grasp, and move his object into its target area. The participant’s GO signal consisted instead in the moment when the confederate lifted his object. If the participant or the confederate started to move before their own GO signal, an error signal appeared at the center of the table, and the trial was discarded (*N* = 270, equal to 7% of the trials). The trial concluded when the participant finally placed his/her object on its target area (Fig. 1b). At this point, after 2,000 ms from the end of the action sequence, both agents heard a lower beeping sound (frequency: 440 Hz; duration: 200 ms), which signaled them to use their left hands to put the objects back to their initial starting areas, and to return to their starting position to receive new instructions.

Participants performed these motor sequences in three different conditions. Importantly, the auditory instructions (i.e., colors) were identical in all three conditions. (I) In the *noninteractive* condition, we told the participants that the goal of the task was *individualistic*: They had to perform *their own* action as quickly and as accurately as possible. At the end of their movement, they received a negative auditory feedback in case they performed their own part of the action sequence too slowly. The feedback consisted in a male voice saying “too slow” and was delivered only when the participants’ movement time was above 2 standard deviations from a reference mean, which we acquired from a pilot experiment (PG mean ± *SD* = 912 ms ± 121 ms; WHP mean ± *SD* = 834 ms ± 113 ms). (II) In the *joint-movement* condition, we told the participants that the goal of the task was *shared* with the confederate: As a pair, they had to perform *the sequence* of actions as quickly and accurately as possible. During this condition, the negative auditory feedback (i.e., male voice saying “too slow”) was delivered when the sum of the movement times of both the confederate’s and the participant’s actions in each sequence was above the relative reference mean acquired from the pilot experiment (PG-PG mean ± *SD* = 1,613 ms ± 159 ms; WHP-WHP mean ± *SD* = 1475 ms ± 149 ms; PG-WHP or WHP-PG mean ± *SD* = 1,570 ms ± 165 ms). (III) In the *joint-outcome* condition, the goal of the task was also referred to as *shared*, but, importantly, it was made to be perceived as a tangible sensory outcome, produced physically in the environment by the two actions performed by the pair. We told participants that, during this condition, each action (i.e., PG or WHP) produced a different colored circle in the environment (i.e., yellow or blue), and that, as a result of this, the combination of the two actions performed by the pair during each sequence produced, at the end of the sequence, a colored circle at the center of the table (Ø = 15 cm). The color of the circle was indeed a direct result of the two types of grasp performed. The circle could in fact be: (i) yellow, if both agents performed the same (i.e., congruent) type of grasp associated with the yellow label (e.g., PG-PG action sequence); (ii) blue, if both agents performed the same (i.e., congruent) type of action associated with the blue label (e.g., WHP-WHP action sequence); (iii) green, if the two agents performed different (i.e., incongruent) types of grasp (i.e., PG-WHP action sequence, or WHP-PG action sequence). In this framework, we told participants that the goal was to produce the final, colored circle as quickly and as accurately as possible. The negative auditory feedback that could be delivered (i.e., male voice saying “too slow”) was computed in the same way as in the *joint movement* condition.

The order of presentation of the conditions was counterbalanced between the participants. During each condition, the participants performed 20 experimental trials for each of the four possible action sequences (i.e., PG-PG, PG-WHP, WHP-PG, WHP-WHP), leading to a total of 80 experimental trials per condition. The total number of trials, and—consequently—of individual movements expected for machine learning data analyses was thus 3,840 (240 trials per 16 participants).

Before each condition, participants performed 10 practice trials. The experimental trials were randomly interspersed with catch trials (20% of total number of trials; i.e., 16 trials per condition), which were designed to maintain the participants’ attention focused on the action performed by the confederate across all trials, and across all conditions. A catch trial appeared identical to an experimental trial, up until the moment when the confederate grasped his object. At that moment in fact, a question mark was projected near the object of the confederate: This instructed the participant to avoid performing the movement, and to just inform the experimenter on whether the action performed by the confederate was the right one or the wrong one, relatively to the instruction that the participant heard at the beginning of the trial. In half of the catch trials, the confederate was—unknowingly—instructed to perform an action that differed from what the participant expected (i.e., wrong action). The entire experiment lasted for approximately 60 minutes. Stimuli presentation and trial randomization were controlled through E-Prime software (Version. 2.0; Psychology Software Tools Inc., Pittsburgh, PA).

### Apparatus

Participants performed the experiment together with a confederate (male, 35 years old). They sat at opposite sides of a table (140 × 120 cm), facing each other (Fig. 1a). A two-layer reinforced honeycomb plastic panel (100 cm × 110 cm) was placed on the center of the table. Four square-shaped cavities were carved out from the first layer of the panel and replaced with four square-shaped sensors. Each sensor was constructed to be sensible to both press and release actions. There were two *small* sensors (4.3 cm × 4.3 cm) and two *large* sensors (9.8 cm × 9.8 cm). The two *small* sensors were placed along the two agents’ midline, 11 cm distant from the two sides of the panel that faced each agent. These sensors were used as the starting point for both the participant’s and the confederate’s actions. The two *large* sensors were placed as follows. One was placed 15-cm distant from the confederate’s right-hand side of the panel, and 35-cm distant from the side of the panel that faced the confederate. The other was placed 15-cm distant from the participant’s right-hand side of the panel, and 50-cm distant from the side of the panel that faced the participant (Fig. 1b).

Two identical objects (height: 13 cm) were placed on the table, 15 cm distant from each agent’s right-hand side of the panel, and 35 cm distant from the side of the panel that faced each agent. The objects were designed to be grasped with either a precision grip (PG), or a whole-hand prehension (WHP). They consisted of two superimposed cylinders with different diameters (upper part: height = 3 cm, Ø = 2.5 cm; lower part: height = 10 cm, Ø = 5.5 cm). In front of the confederate’s object, a square-shaped target (9.8 cm × 9.8 cm) was drawn on the panel and represented the confederate’s action target area. The participant’s target area coincided with the *large* sensor placed in front of her/him.

A projector was positioned on the ceiling above the table and was used to deliver visual stimuli on the panel. Both the participant and the confederate were outfitted with headphones, through which they received auditory instructions and signals.

### Kinematic recording

Movement kinematics were recorded using a near-infrared camera motion-capture system (frame rate: 100 Hz; Vicon Nexus v.2.5). Eight cameras were placed in a semicircle at a distance of 1.5–2 m from the table where the two agents were seated.

Both agents’ right hands were outfitted with 20 retro-reflective hemispheric markers (6 mm in diameter). Data analyses were performed on the kinematic profile of the markers placed on the tip of the thumb, on the tip of the index finger, and on the radial aspect of the wrist. Four additional markers were placed on each of the two objects used.

### Data processing

After data collection, each trial was individually inspected for correct marker identification and then run through a low-pass Butterworth filter with a 8-Hz cutoff. Trials in which the quality of marker reconstruction was poor (*N* = 79, equal to 2% of the trials) and trials in which either the confederate or the participant performed wrong or inaccurate movements (*N* = 142, equal to 4% of the trials) were discarded from the dataset and not considered for further analyses. For data processing and analysis, a MATLAB custom script (MATLAB; MathWorks, Natick, MA) was used to compute the variables of interest. Each variable was computed within the time window from onset to offset of the reach-to-grasp phase of the movement. For both agents, movement onset was defined as the first time point at which the velocity of the wrist crossed a 20-mm/s threshold. Movement offset was defined as the time point, within 10 ms before and 10 ms after the object started to be lifted, at which the velocity of the wrist was at the lowest. Within these time windows, we computed for both agents the following kinematic features:

- Wrist Velocity (WV), defined as the module of wrist velocity (mm/s);
- Wrist Acceleration (WA), defined as the rate of change of wrist velocity (mm/s^2^);
- Wrist Jerk (WJ), defined as the rate of change of the module of wrist acceleration (mm/s^3^);
- Grip Aperture (GA), defined as the distance between the marker placed on the tip of the thumb and the marker placed on the tip of the index finger (mm);
- Wrist Height (WH), defined as the z-component of the marker placed on the wrist (mm).

Each of these variables was expressed with respect to normalized (%) rather than absolute (ms) duration, and was then resampled at intervals of 10% of the normalized movement time. In other words, once movement onset and movement offset were established, we resampled each kinematic variable (e.g., WV) in 10 time intervals to obtain the value of each variable at different time points of the movement, from 10% to 100% of movement time. This fine-grained description proved to be more sensitive to catch subtle kinematic differences than solely relying on few kinematic landmarks like peaks (Montobbio et al., 2022; Patri et al., 2020; Soriano et al., 2018).

In order to have a direct comparison of the visuo-motor interference effect as measured in the previous literature, we also computed the following variables:

- Performance Accuracy, defined as the correctness or wrongness of the performed action, compared with the instructed one (values: 1, 0);
- Reaction Time (RT), defined as the interval between the participant’s GO signal and the onset of their reach-to-grasp movement (ms);
- Movement Time (MT), defined as the interval between onset and offset of the reach-to-grasp movement performed by the participants (ms).

### Data analyses

Analyses on RT and MT were performed separately using two linear mixed-effect models, where we considered as fixed effects Congruency (*congruent*, *incongruent*), and Condition (*noninteractive*, *joint movement*, *joint outcome*), together with their interactions. By-subjects random intercepts were included to account for between-subject variability. Holm-Bonferroni correction was applied to correct for multiple pairwise comparisons.

The level of attention paid by participants during the three conditions was assessed by measuring participants’ response accuracy during catch trials. Given the ceiling performance (i.e., not normal distribution), the nonparametric Wilcoxon rank sum test was used to evaluate differences between conditions.

For what concerns the machine learning analyses, the dataset used consisted of the 3,349 reach-to-grasp movements performed by the participants. This dataset was divided into two subsets depending on the type of grasp performed by the participants (i.e., PG or WHP). The PG subset consisted of 1651 reach-to-grasp movements. The WHP subset consisted of 1698 reach-to-grasp movements. All the analyses reported below have been performed on the PG subset and then replicated on the WHP subset.

### Quantification of visuo-motor interference during noninteractive, joint-movement, and joint-outcome conditions

To quantify the presence of visuo-motor interference, we used a machine learning approach. The kinematic features of participants’ reach-to-grasp movements were used as predictors to classify the action performed by the confederate. The confederate’s action could be *congruent* (when, compared with the participant, he performed the same type of grasp) or *incongruent* (when, compared with the participant, he performed a different type of grasp). To investigate the modulation of visuo-motor interference between the *noninteractive*, the *joint-movement* and the *joint-outcome* conditions, we trained, validated, and tested, for each experimental condition, 1,000 support vector machines regularized with least absolute shrinkage and selection operator (Tibshirani, 1996). SVMs are supervised learning algorithms that seek to find the optimal hyperplane that separates a dataset into classes. The objective of the SVM algorithm is to minimize the hinge loss function, while the LASSO regularizer allows to maximize interpretability and generalizability of the model by increasing sparsity in the model coefficients. In order to maintain the same number of trials in each SVM-LASSO model of each condition, for each of the 1,000 iterations we randomly selected 480 trials (i.e., 30 trials per participant: 15 *congruent* trials and 15 *incongruent* trials). Data splitting was then performed by employing a 15-fold cross-validation procedure. K-fold cross-validation involves splitting the dataset into equally sized folds. During each iteration, the model is trained on k-1 folds, and then tested on the fold left out. We repeated this procedure 15 times, each time using a different fold as the testing dataset, and the other 14 folds as the training dataset. Each of the 15 folds contained 32 trials (i.e., two trials per participant: one *congruent trial* and one *incongruent trial*). Hyper-parameter was recursively tuned on all but one fold of the training set by implementing a nested 14-fold cross-validation procedure. Classification accuracy was used as a measure of classification performance. To test whether the classification accuracy significantly exceeded chance level, we randomly permuted the *congruent*/*incongruent* labels (1,000 permutations per condition) and recomputed the classification accuracy after each permutation. This allowed us to obtain an empirical null distribution of *random* classification accuracies. The empirical *p* values were then determined as the proportion of times that the classification accuracy obtained from the random permutations exceeded the average classification accuracy obtained from the original classifiers (Ojala & Garriga, 2010).

To test the difference in classification accuracy between experimental conditions, we computed empirical *p* values, which were determined as the proportion of times that the classification accuracy obtained from the classifiers of one condition was lower than the average classification accuracy obtained from the classifiers of another condition. Holm–Bonferroni correction was applied to correct for multiple pairwise comparisons.

The level of significance, α, was set at 0.05 for all statistical comparisons.

### Quantification of visuo-motor interference in single kinematic features across conditions

To investigate whether each kinematic feature encoded information about the action performed by the confederate, we performed 1,000 repetitions of five separate SVM-LASSO models for each experimental condition. Each model was trained and tested using only one kinematic feature at a time (i.e., WV, WA, WJ, GA and WH). For each model, we reproduced the same k-fold cross-validation procedure described above (i.e., 15 folds; 32 trials per fold; in each fold, two trials per participant: one *congruent*, one *incongruent*). Hyper-parameter was recursively tuned on all but one fold of the training set by implementing a nested 14-fold cross-validation procedure. To test whether the classification accuracy significantly exceeded chance level, we randomly permuted the *congruent*/*incongruent* labels (1,000 permutations per kinematic feature per condition) and recomputed the classification accuracy after each permutation. The *p* values were determined as the proportion of times that the classification accuracy obtained from the random permutations exceeded the average classification accuracy obtained from the original classifiers.

The difference in classification accuracy between experimental conditions was tested, for each kinematic feature, by computing empirical *p* values, determined as the proportion of times that the classification accuracy obtained from the classifiers of one condition was lower than the average classification accuracy obtained from the classifiers of another condition. Holm–Bonferroni correction was applied to correct for multiple pairwise comparisons.

The level of significance, α, was set at 0.05 for all statistical comparisons.

### Specificity and temporal evolution of the visuo-motor interference effect

To gain further understanding on whether and how the spatiotemporal kinematic parameters expressed by the confederate were embodied by participants through time, we computed, only for the kinematic features that were found significantly discriminative, a measure of *kinematic distance* between the participants and the confederate. For each *incongruent* trial of the *noninteractive* condition, we measured, within each kinematic feature, the Euclidean distance between the kinematic profile displayed by the participant and the average kinematic profile displayed by the confederate in that particular action sequence. Each *kinematic distance* was thus computed as the Euclidean distance between two 10-value vectors (i.e., the kinematic profile displayed by the confederate, and the kinematic profile displayed by the participant, each expressed through 10 time intervals, from 10% to 100% of movement time). We focused on the *noninteractive* condition as it was the one in which the effect of visuo-motor interference was significantly more pronounced. For each kinematic feature, the measure of *kinematic distance* was then analyzed by using a linear mixed effect model where we considered as fixed effect the trials within condition (*first half*, *second half*). By-subjects random intercepts were included to account for between-subject variability. We expected to observe, for each kinematic feature, a main effect of Trials within condition, for which the *kinematic distance* would have been lower in the second half of the trials, compared with the first half. This would indicate the participant’s kinematics become more similar to the confederate’s over time. We applied the false discovery rate method (Benjamini & Hochberg, 1995) to correct the *p* values obtained, in order to allow a comparison between the effect obtained in all the analyses performed (i.e., for each kinematic feature).

## Results

The average performance accuracy of participants was 0.996 (*SEM* = 0.001).

The linear mixed-effect model performed on RT revealed a significant main effect of congruency, *F*(1, 3322.02) = 8.969; *p* = 0.003, which showed that participants were significantly slower in incongruent as compared with congruent trials (*p* = 0.003). There was also a significant main effect of condition, *F*(2, 3322.04) = 11.649; *p* < 0.001, which showed that participants were significantly faster during the *noninteractive* than both the *joint-movement* (*p* = 0.003) and the *joint-outcome* conditions (*p* < 0.001).

The linear mixed-effect model performed on MT revealed a significant main effect of congruency, *F*(1, 3321.02) = 10.075; *p* = 0.002, which showed that participants performed slower movements during incongruent as compared with congruent trials (*p* = 0.002). This effect was modulated by the condition, as shown by the significant interaction effect, *F*(2, 3321.02) = 3.089; *p* = 0.046: movements in incongruent trials were significantly slower than in congruent trials during the *noninteractive* condition (*p* = 0.011) but not during the *joint movement* (*p* > 0.999) nor the *joint outcome* conditions (*p* = 0.195). Group mean values of Performance Accuracy, RT and MT, and detailed results of the analyses on RT and MT can be accessed in the Supplementary Results.

The average response accuracy to catch trials was 0.97 (*SEM* = 0.007). There was no difference between the response accuracies of the *noninteractive*, the *joint-movement*, and the *joint-outcome* conditions (*p*s from 0.40 to 0.90).

All analyses reported below were performed separately on participants’ PG actions and on participants’ WHP actions. Here, we present the results obtained using PG actions as predictors. The results concerning WHP actions can be accessed in the Supplementary Results.

### Quantification of visuo-motor interference during noninteractive, joint-movement, and joint-outcome conditions

**Fig. 2:**
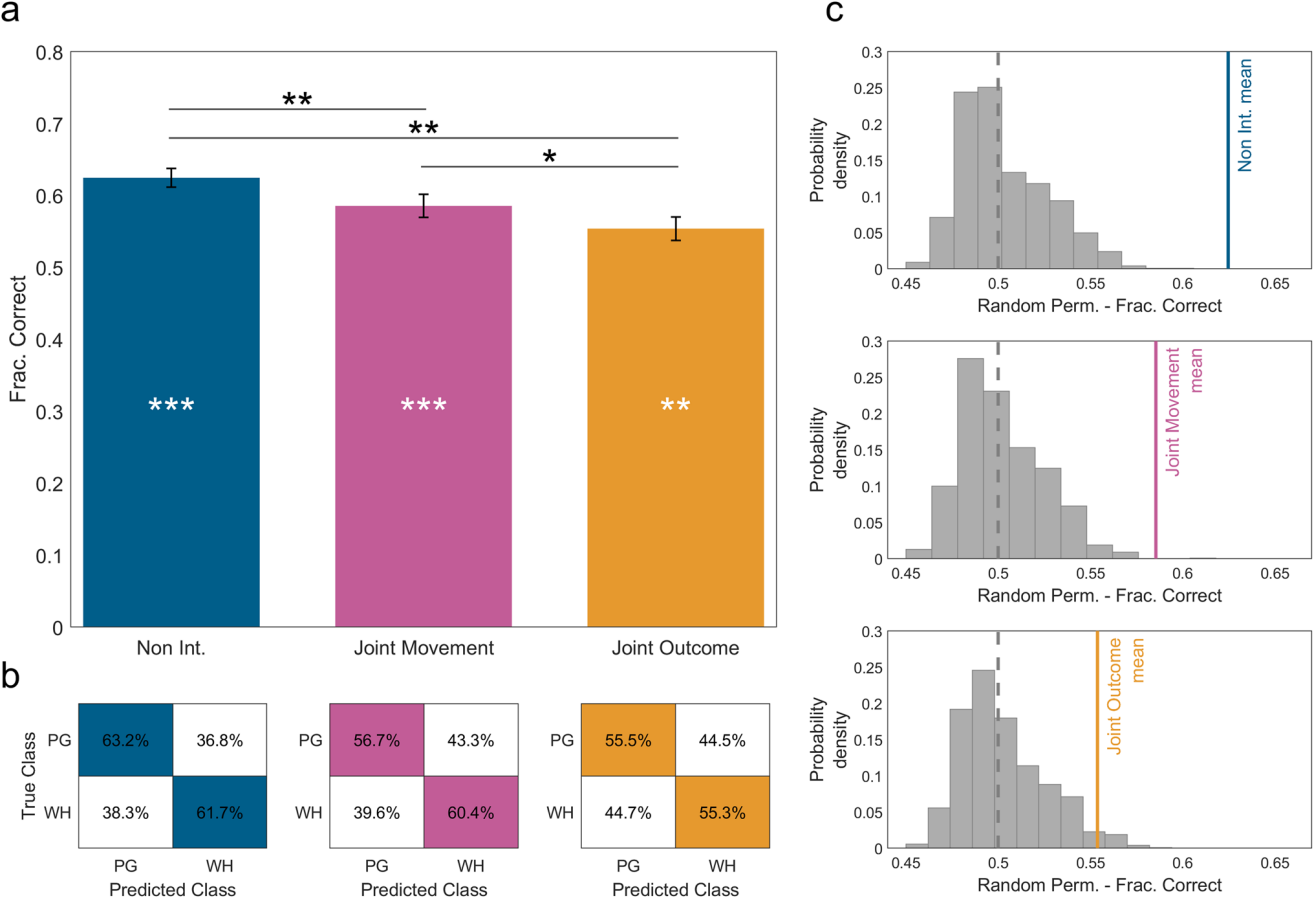
Classification accuracy during noninteractive, joint movement, and joint outcome conditions. Panel (**a**) shows a bar plot representing the mean classification accuracies of the 100 SVM-LASSO models performed for each condition. Bars indicate standard deviation (*SD*). White asterisks denote significant (i.e., above chance) classification accuracies (*p < 0.05; **p < 0.01; ***p < 0.001). Black asterisks denote significant differences between conditions (*p < 0.05; **p < 0.01). Panel (**b**) shows the confusion matrices corresponding to each condition (rows are the true classes). The three histograms of panel (**c**) represent, for each condition, the empirical distribution of the classification accuracies obtained from the 1000 SVM-LASSO models computed after the random permutation of labels. For each of the three histograms, the solid line represents the mean classification accuracy obtained from the original models of the corresponding condition. The dashed line indicates 0.5 chance level

Classification results revealed that participants’ kinematics enabled to classify the action performed by the confederate (i.e., *congruent/incongruent*) significantly above the level of chance in all three conditions (*noninteractive* mean ± *SEM* = 0.625 ± 0.001, empirical-*p* = 0.001; *joint-movement* mean ± *SEM* = 0.586 ± 0.001, empirical-*p* = 0.002; *joint-outcome* mean ± *SEM* = 0.554 ± 0.001, empirical-*p* = 0.032). This indicates that, in all conditions, the action performed by the confederate was interfering with the participants’ movement kinematics. However, visuo-motor interference was significantly more pronounced in the *noninteractive* condition, as compared with both the *joint-movement* condition (empirical-*p* = 0.008) and the *joint-outcome* condition (empirical-*p* = 0.003). Classification accuracy was also significantly higher in the *joint-movement* condition, compared with the *joint-outcome* condition (empirical*-p* = 0.024; Fig. 2).

### Quantification of visuo-motor interference in single kinematic features across experimental conditions

Classification results revealed that, during the *noninteractive* condition, the kinematic features that, per se, could significantly encode the confederate’s action were *Wrist Velocity* (*WV*; mean ± *SEM* = 0.568 ± 0.001, empirical-*p* = 0.003), *Wrist Acceleration* (*WA*; mean ± *SEM* = 0.562 ± 0.001, empirical-*p* = 0.014), *Grip Aperture* (*GA*; mean ± *SEM* = 0.569 ± 0.001, empirical-*p* = 0.002), and *Wrist Height* (*WH*; mean ± *SEM* = 0.585 ± 0.001, empirical*-p* = 0.001). Similar results were obtained during the *joint movement* condition, where *WV* (mean ± *SEM* = 0.550 ± 0.001, empirical-*p* = 0.019), *WA* (mean ± *SEM* = 0.561 ± 0.001, empirical*-p* = 0.003), *GA* (mean ± *SEM* = 0.559 ± 0.001, empirical-*p* = 0.010), and *WH* (mean ± *SEM =* 0.553 ± 0.001, empirical-*p* = 0.004) significantly encoded the confederate’s action. During the *joint-outcome* condition, none of the models using single kinematic features could significantly predict the actions performed by the confederate (*WV* mean ± *SEM* = 0.511 ± 0.001; *WA* mean ± *SEM* = 0.510 ± 0.001; *GA* mean ± *SEM* = 0.541 ± 0.001; *WH* mean ± *SEM* = 0.522 ± 0.001; empirical-*p*s = 0.256, 0.309, 0.051, and 0.168, respectively). For all conditions, the models that used *Wrist Jerk (WJ)* as predictor did not perform above chance level (empirical-*p*s ranging from 0.055 to 0.512).

**Fig. 3:**
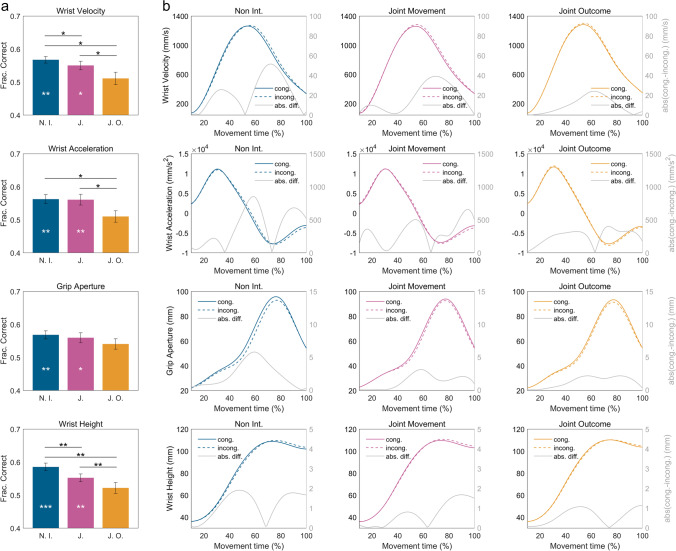
Classification accuracy and kinematic profiles of single kinematic features during each condition. Panel (**a**) shows, for each of the four relevant (i.e., significantly discriminative) kinematic features, a bar plot representing the mean classification accuracies of the 1000 SVM-LASSO models performed for each condition. Bars indicate standard deviation (*SD*). White asterisks denote significant (i.e., above chance) classification accuracies (*p < 0.05; **p < 0.01; **p < 0.001). Black asterisks denote significant differences between conditions (*p < 0.05; **p < 0.01). Panel (**b**) represents, for each of these kinematic features, the mean kinematic profiles displayed by participants while performing PG actions on *congruent* and *incongruent* trials, during the *noninteractive*, the *joint movement*, and the *joint outcome* conditions. In each plot, the grey line represents the absolute difference between the mean kinematic profile displayed during *congruent* trials and the mean kinematic profile displayed during *incongruent* trials. For each kinematic feature, the kinematic difference between congruent and incongruent trials is visibly higher during the *noninteractive* condition, compared with the *joint movement* and the *joint outcome* condition

Classification accuracy of models trained on single kinematic features was then compared between conditions. As shown in Fig. 3, the classification accuracy was systematically higher in the *noninteractive* compared with *joint-outcome* condition (all empirical-*p*s < 0.05). With the exception of *GA*, similar results were obtained when comparing the *joint-movement* with the *joint-outcome* condition (empirical-*p*s ranging from 0.002 to 0.03). Significant differences were also found between the *noninteractive* and the *joint movement* condition. For both *WV* and *WH,* the classification accuracy was higher in the former (empirical-*p*s = 0.049 and 0.004, respectively).

### Specificity and temporal evolution of the visuo-motor interference effect

**Fig. 4:**
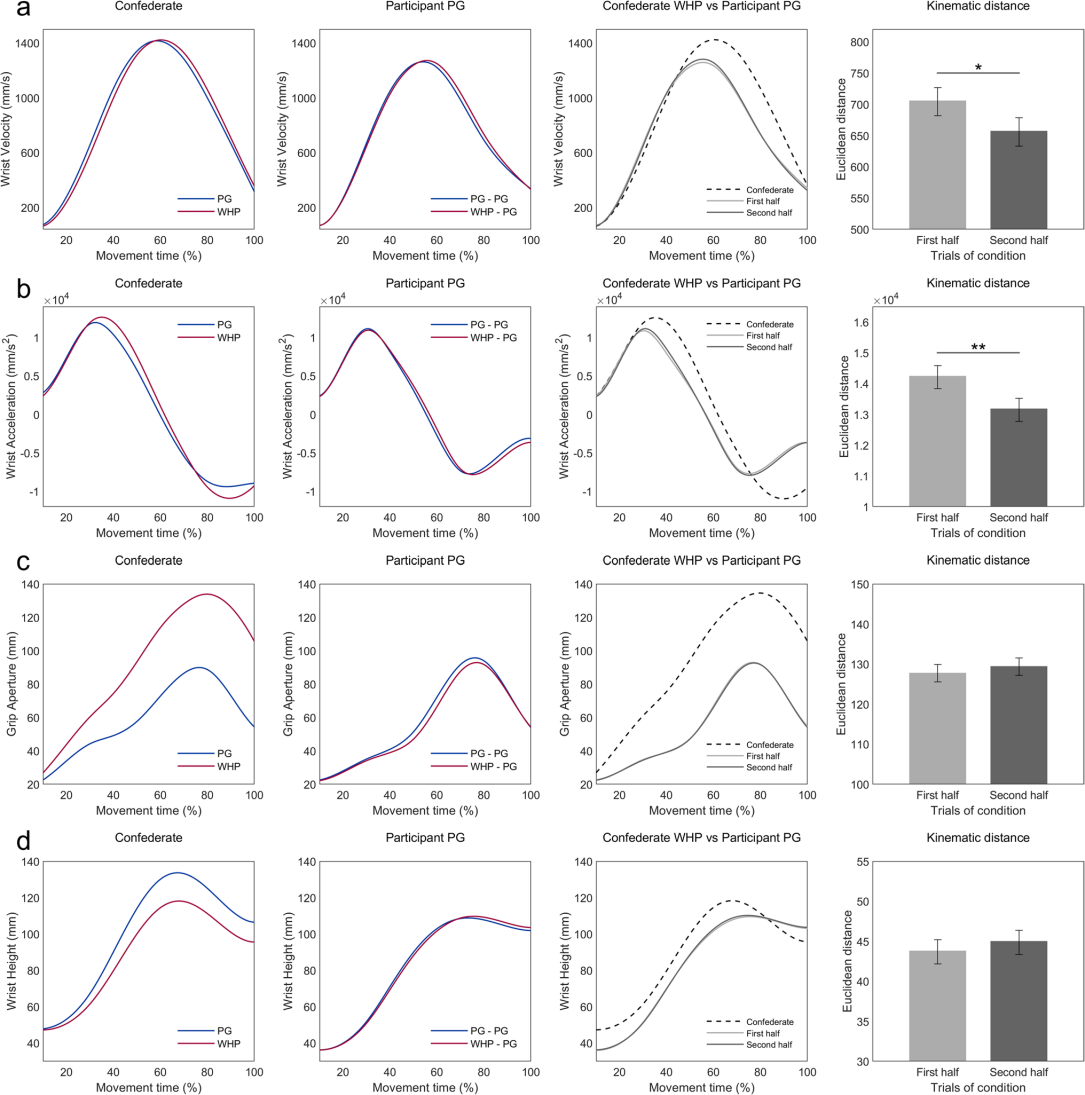
Kinematic distance between confederate and participant. The graphs in the first column represent the mean kinematic profiles of Wrist Velocity (**a**), Wrist Acceleration (**b**), Grip Aperture (**c**), and Wrist Height (**d**), displayed by the confederate while performing PG or WHP actions during the *noninteractive* condition. The graphs in the second column represent, for each kinematic feature, the mean kinematic profile displayed by participants while performing PG actions during *congruent* trials (PG-PG motor sequence) or during *incongruent* trials (WHP-PG motor sequence) of the *noninteractive* condition. The graphs in the third column represent, for each kinematic feature, the mean kinematic profile displayed by the confederate while performing a WHP action (dotted line), and the mean kinematic profiles displayed by the participants in the first half (light grey) and second half (dark grey) of the trials of the *noninteractive* condition, while performing a PG action during *incongruent* trials (i.e. after observing the confederate perform a WHP action). The bar plots in the fourth column represent, for each kinematic feature, the Euclidean distance between the participants’ and the confederate’s kinematic profiles, during the first half and the second half of the *incongruent* trials of the *noninteractive* condition. Bars indicate standard error (*SE*). Asterisks denote significant differences (*p < 0.05; **p < 0.01)

Participants’ *WV* and *WA* displayed a pattern of similarity to the action performed by the confederate, as if, on these variables, observing a PG action elicited the kinematic properties related to a PG action, while observing a WHP action elicited the kinematic properties related to a WHP action (Fig. 4a and b). Instead, *GA* and *WH* displayed a pattern of complementarity to the action performed by the confederate, as if, on these variables, observing a PG action elicited the kinematic properties related to a WHP action, while observing a WHP action elicited the kinematic properties related to a PG action (Fig. 4c and d). The analysis on the Euclidean distance between the participants’ and the confederate movements (i.e., *kinematic distance*) revealed that these two distinct kinematic modulations patterns also displayed two distinct modulation patterns *through time*. On *WV* and *WA*, participants showed a decreased *kinematic distance* from the confederate in the second half of the experimental trials, WV Distance mean ± *SEM* = 643.39 ± 17.29.; WA Distance mean ± *SEM* = 12492.01 ± 300.73), compared with the first half (WV Distance mean ± *SEM* = 701.20 ± 17.91, *F*(1, 247.08) = 5.632, *p* = 0.036; WA Distance mean ± *SEM* = 13585.86 ± 304.84, *F*(1, 247.03) = 10.164, *p* = 0.008 (Fig. 4a and b). These two kinematic features thus displayed a pattern of increased similarity to the kinematics of the confederate through time. Instead, GA and WH revealed no such modulation, GA Distance – first half mean ± *SEM* = 113.17 ± 1.86; second half mean ± *SEM* = 114.08 ± 2.14, *F*(1, 247.07) = 0.842, *p* = 0.477; WH Distance – first half mean ± *SEM* = 127.20 ± 2.34; second half mean ± *SEM* = 122.04 ± 2.55, *F*(1, 245.05) = 0.507, *p* = 0.477 (Fig. 4c and d).

## Discussion

In the present study, we investigated how interactivity modulates the effect of visuo-motor interference. The aim was to understand how our motor system is affected by the observation of actions performed by others when the level of interactivity required to accomplish a task changes.

Using the kinematic features of the participants’ movements, we were able to classify, in each of our three conditions, the action performed by the confederate. This means that, as a result of visuo-motor interference, the same type of movement was performed differently, depending on what action the confederate had just performed. Crucially, the magnitude of this interference was significantly more pronounced during the *noninteractive* condition, compared with the two joint-action conditions (Fig. 2). These results, obtained from the analysis of participants’ PG movements, and fully replicated on participants’ WHP movements (see Supplementary Results), reveal that, compared with a noninteractive scenario, the observation of a partner’s action becomes less disruptive when we share a goal with the him/her. This is true regardless of whether the common goal requires to produce a concrete sensory outcome (*joint-outcome* condition) or only a joint movement configuration with the other (*joint-movement* condition). Indeed, although we found a significantly reduced visuo-motor interference in the *joint-outcome* compared with the *joint-movement* condition, this result was not robust, as it was present only in participant’s PG movements.

Our results on RTs replicate previous findings on congruency-dependent modulations (for a meta-analysis, see Cracco et al., 2018). These data showed that, overall, participants were slower during incongruent trials compared with congruent ones. The analysis of MTs also revealed that the congruency effect was modulated by the experimental condition: incongruent trials were significantly slower than congruent ones in the *noninteractive* condition but not in the *joint-movement* nor the *joint-outcome* condition. Importantly, this latter result replicates previous findings (Clarke et al., 2019; Sacheli, Arcangeli, & Paulesu, 2018a; Sacheli, Verga, et al., 2019b), while evidence for a reduced visuo-motor interference in the *joint-movement* condition is new but anyway strongly supported by our kinematic data analysis. Indeed, our novel analytical approach shows that the reduction in visuo-motor interference in both Joint conditions is explicitly visible at single trial level in the kinematic unfolding of reach-to-grasp movements.

The results of our study are in line with the hypothesis that joint action planning is based on dyadic motor plans, in which both our own and our partner’s actions are represented in terms of their predicted effects in the environment (Sacheli, Arcangeli, & Paulesu, 2018a). Within this view, representing a joint goal (shared between partners) triggers strong expectations on what actions the partner will do to contribute to its achievement (Pesquita et al., 2018). As a consequence, the partner’s actions do not need to be simulated (as it happens in noninteractive social contexts during automatic imitation), but only monitored, to see if they meet the expectations (see also studies on action monitoring during joint actions; Boukarras et al., 2022; Moreau et al., 2020, 2022; Sacheli et al., 2021, 2022). This view thus suggests that, during joint actions, the motor system shifts from the automatic simulation of an observed action to the active prediction of its consequences, to monitor if the joint goal will be achieved. Our results reinforce this hypothesis by showing that visuo-motor interference (i.e., a behavioral effect associated with automatic imitation) was stronger in the *noninteractive* condition compared with the joint action conditions (*joint movement* and *joint outcome*). Possibly, this was due to the shift from action simulation to action prediction, as suggested by the dyadic motor plan hypothesis. Importantly however, this evidence is only indirect, as we did not measure any index of predictive processes or brain activity in the present study.

Interestingly, in our study visuo-motor interference was reduced not only when the movement of the other was relevant in terms of what it would have produced in the environment (*joint-outcome* condition), but also when the movement of the other was just relevant ‘per se’ to achieve the common goal (*joint-movement* condition). These findings differ from those of della Gatta et al. (2017), who reported increased visuo-motor interference in a joint movement-like experimental paradigm. Reasons for this discrepancy may lie in the between-group design and in the type of movements performed in della Gatta et al. (2017), which were continuous, rhythmic, and synchronous, differently from the present study. These differences may not allow for a direct comparison between the two studies. Further research is thus needed to explore specifically in which conditions the effect of visuo-motor interference could be enhanced.

Our results support the hypothesis that, as long as the partner’s actions are integrated within an overarching dyadic motor plan, they interfere less, even if they represent a pure motor contribution to achieve the desired shared goal and cannot be represented in terms of their predicted sensory outcomes in the environment. This interpretation does not deny the predictive nature that these action representations might have. On the contrary, our results support the idea that, during joint actions, the action performed by the other is indeed processed in predictive terms. However, these predictions may not be solely contingent upon the production of an outcome in the environment. They may also pertain to the movement itself. Thus, during joint action, our predictions may vary and involve the movement executed by others and/or the outcome resulting from that movement (Pesquita et al., 2018; Sacheli, Meyer, et al., 2019a). The specific level of the motor hierarchy that becomes crucial for motor predictions might depend on which level is critical to achieve the joint goal. This interpretation is supported by previous evidence for a correlation between joint performance and reduced visuo-motor interference (Sacheli et al., 2013) and for a causal role of brain regions responsible for predictive coding of others’ actions in supporting joint performance (Era et al., 2018; Hadley et al., 2015; Sacheli et al., 2015; Sacheli, Tieri, et al., 2018b).

It is, however important to underline that our results can only account for the visuo-motor interference that arises during cooperative social interactions, examined in the context of joint actions. Future studies should investigate whether and how the effect of visuo-motor interference changes also depending on other interactivity scenarios (e.g., during competitive interactions).

It is worth noting that our results cannot be simply explained by an attentional difference between conditions. In fact, during each condition, experimental trials were randomly interspersed with catch trials designed to maintain the participants’ attention focused on the movement performed by the confederate. The response accuracy derived from the analysis of catch trials allows us to exclude the presence of any difference in attentional focus between conditions. Moreover, performance accuracy was at ceiling in all experimental conditions (see Supplementary Results), suggesting that there was no difference in task difficulty across the three experimental conditions, which were identical in terms of perceptual features, motor requirement, and even available information about the partner’s behavior: The only element differentiating the *noninteractive*, *joint-movement*, and *joint-outcome* conditions was the instructions that stressed the independent versus shared nature of the agent’s goal, and the presence or not of an outcome as consequence of the motor sequence. It is however important to underline that there might have been a difference in the perceived complexity between the two joint-action conditions and the *noninteractive* one. Indeed, although we found no difference in performance accuracy between conditions, the analysis on reaction times revealed that responses were significantly faster during the *noninteractive* condition, compared with both the *joint-movement* and the *joint-outcome* conditions. Participants might thus have perceived the joint-action conditions as implicitly more difficult than the *noninteractive* one, although this did not affect the correctness of their responses.

For what concerns the specificity and the temporal evolution of the visuo-motor interference effect, our results are ambiguous. Indeed, while some kinematic features clearly displayed a pattern of increased similarity to the kinematic unfolding presented by the confederate, other features did not display this expected effect.

In particular, we found that participants’ velocity and acceleration profiles showed a pattern of *similarity* to the observed action (as if observing a PG action triggered the kinematic profile of a PG action, while observing a WHP action triggered the kinematic profile of a WHP action; Fig. 4a and b). Furthermore, when looking at the *kinematic distance* between the participants and the confederate in the first and in the second half of the experimental trials, results show that within these features the similarity increased over the course of the experiment. This supports the idea of an interference not, or at least not only, driven by a high-level representation of the other’s task, which, in case of incongruence, may generically impact on participant’s action. Such interference seems instead linked to the specific kinematic properties of the observed action (Hardwick & Edwards, 2011), as participants were increasingly converging towards the kinematic profile displayed by the confederate (Rocca & Cavallo, 2021).

Participants’ grip aperture and wrist height profiles showed instead a pattern of *complementarity* to the observed action (as if observing of a PG action triggered the kinematic profile of a WHP action, while observing a WHP action triggered the kinematic profile of a PG action; Fig. 4c and d). This effect can be explained by the fact that, during the experiment, participants observed not only the *movement* performed by the confederate but also the *object* manipulated by him. At the moment of grasp, participants observed the confederate’s hand grasping *one part* of the object (i.e., bottom large part or top small part): the other part of the object remained visible and, ideally, graspable. This visible left-out part of the object might have acted as a *distractor* object for participants, eliciting the relative grip (PG or WHP) in those specific kinematic parameters that are known to be highly influenced by the properties of a to-be-grasped object (Castiello, 1999; Tipper et al., 1997). This interpretation is also supported by the absence of any reduction of kinematic distance between the agents over the course of the experimental trials (Fig. 4c and d). The kinematic modulation displayed by these features was thus unrelated to the movement kinematics exhibited by the confederate.

## Conclusion

Results of the present study show how, in the context of joint actions, task interactivity can change not only the way we perceive the actions performed by others, but also the effects they have on our own motor responses. Observing others’ movements affects us deeply, and this effect is mediated by the significance and the relevance that the observed action has to our social interaction. Actions are processed and produce a motor response that varies as a function of the social context in which they are embedded.

### Supplementary Information

ESM 1 (DOCX 30 kb)
