# Supplementary material for: Visuo-motor interference is modulated by task interactivity: A kinematic study
Source: Psychon Bull Rev. 2023 May 1;30(5):1788–801. doi: 10.3758/s13423-023-02297-z (PMC10716078; doi:10.3758/s13423-023-02297-z)
Supplement: Supplementary file 1 — (DOCX 30 kb) [file 13423_2023_2297_MOESM1_ESM.docx]

**Supplementary Results**

**Performance Accuracy, Reaction Time and Movement Time**

**Performance Accuracy**

|  | **congruency** | |
| --- | --- | --- |
| **condition** | *congruent* | *incongruent* |
| *Non Interactive* | 0.998 ± 0.002 | 0.994 ± 0.003 |
| *Joint Movement* | 0.997 ± 0.002 | 0.998 ± 0.002 |
| *Joint Outcome* | 1.000 ± 0.000 | 0.990 ± 0.004 |

In each cell: mean ± SEM

**Reaction Time (ms)**

|  | **congruency** | |
| --- | --- | --- |
| **condition** | *congruent* | *incongruent* |
| *Non Interactive* | 177.65 ± 3.62 | 185.95 ± 3.78 |
| *Joint Movement* | 190.29 ± 4.05 | 195.05 ± 3.90 |
| *Joint Outcome* | 194.56 ± 4.48 | 202.74 ± 4.44 |

In each cell: mean ± SEM

**Movement Time (ms)**

|  | **congruency** | |
| --- | --- | --- |
| **condition** | *congruent* | *incongruent* |
| *Non Interactive* | 379.61 ± 1.91 | 386.22 ± 1.80 |
| *Joint Movement* | 386.67 ± 1.96 | 386.82 ± 1.78 |
| *Joint Outcome* | 379.15 ± 1.90 | 383.48 ± 1.89 |

In each cell: mean ± SEM

**Linear Mixed Effect Models**

Results on Reaction Times

| **effect** | **F** | **df** | **p-value** |
| --- | --- | --- | --- |
| congruency | 8.969 | 1, 3322.02 | 0.003** |
| condition | 11.649 | 2, 3322.04 | 0.000*** |
| congruency by condition | 0.418 | 2, 3322.02 | 0.659 |

(*p < 0.05; **p < 0.01, ***p < 0.001)

Results on Movement Times

| **effect** | **F** | **df** | **p-value** |
| --- | --- | --- | --- |
| congruency | 10.075 | 1, 3321.02 | 0.002** |
| condition | 9.580 | 2, 3321.04 | 0.000*** |
| congruency by condition | 3.089 | 1, 3321.02 | 0.046* |

(*p < 0.05; **p < 0.01, ***p < 0.001)

**Results of analyses performed on WHP subset**

**Quantification of visuo-motor interference during *Non Interactive*, *Joint Movement* and *Joint Outcome* conditions**

Results of SVM-LASSO models performed using WHP actions as predictors:

| **condition** | **mean ± SEM** | **p-value (permutation test)** |
| --- | --- | --- |
| *Non Interactive* | 0.589 ± 0.001 | 0.001** |
| *Joint Movement* | 0.558 ± 0.001 | 0.001** |
| *Joint Outcome* | 0.557 ± 0.001 | 0.001** |

(*p < 0.05; **p < 0.01)

Comparison between conditions:

| **condition(1)** | **condition(2)** | **Holm-Bonferroni corrected empirical p-value** |
| --- | --- | --- |
| *Non Interactive* | *Joint Movement* | 0.048* |
| *Non Interactive* | *Joint Outcome* | 0.048* |
| *Joint Movement* | *Joint Outcome* | 0.476 |

(*p < 0.05; **p < 0.01)

**Quantification of visuo-motor interference in single kinematic features across conditions**

Results of SVM-LASSO models performed using single kinematic features of WHP actions as predictors:

| **condition** | **kinematic feature** | **mean ± SEM** | **p-value (permutation test)** |
| --- | --- | --- | --- |
| *Non Interactive* | *Wrist Velocity* | 0.594 ± 0.001 | 0.002** |
|  | *Wrist Acceleration* | 0.586 ± 0.001 | 0.001** |
|  | *Wrist Jerk* | 0.575 ± 0.001 | 0.001* |
|  | *Grip Aperture* | 0.522 ± 0.001 | 0.156 |
|  | *Wrist Height* | 0.546 ± 0.001 | 0.019* |
| *Joint Movement* | *Wrist Velocity* | 0.553 ± 0.001 | 0.011* |
|  | *Wrist Acceleration* | 0.544 ± 0.001 | 0.034* |
|  | *Wrist Jerk* | 0.539 ± 0.001 | 0.079 |
|  | *Grip Aperture* | 0.512 ± 0.001 | 0.275 |
|  | *Wrist Height* | 0.533 ± 0.001 | 0.059 |
| *Joint Outcome* | *Wrist Velocity* | 0.552 ± 0.001 | 0.012* |
|  | *Wrist Acceleration* | 0.553 ± 0.001 | 0.014* |
|  | *Wrist Jerk* | 0.549 ± 0.001 | 0.020* |
|  | *Grip Aperture* | 0.508 ± 0.001 | 0.295 |
|  | *Wrist Height* | 0.540 ± 0.001 | 0.020* |

(*p < 0.05; **p < 0.01)

Comparison between conditions:

| **kinematic feature** | **condition(1)** | **condition(2)** | **Holm-Bonferroni corrected empirical p-value** |
| --- | --- | --- | --- |
| *Wrist Velocity* | *Non Interactive* | *Joint Movement* | 0.009** |
|  | *Non Interactive* | *Joint Outcome* | 0.009** |
|  | *Joint Movement* | *Joint Outcome* | 0.412 |
| *Wrist Acceleration* | *Non Interactive* | *Joint Movement* | 0.006** |
|  | *Non Interactive* | *Joint Outcome* | 0.014* |
|  | *Joint Movement* | *Joint Outcome* | 0.751 |
| *Wrist Jerk* | *Non Interactive* | *Joint Movement* | 0.003* |
|  | *Non Interactive* | *Joint Outcome* | 0.088 |
|  | *Joint Movement* | *Joint Outcome* | 0.735 |
| *Grip Aperture* | *Non Interactive* | *Joint Movement* | 0.623 |
|  | *Non Interactive* | *Joint Outcome* | 0.623 |
|  | *Joint Movement* | *Joint Outcome* | 0.518 |
| *Wrist Height* | *Non Interactive* | *Joint Movement* | 0.354 |
|  | *Non Interactive* | *Joint Outcome* | 0.639 |
|  | *Joint Movement* | *Joint Outcome* | 0.773 |

(*p < 0.05; **p < 0.01)

**Embodiment of the confederate’s kinematic profile through time**

Results of linear mixed effect models performed on the kinematic distance computed on *incongruent* WHP actions of the *Non Interactive* condition.

| **kinematic feature** | **trials of condition** | **mean ± SEM** | **F** | **df** | **FDR corrected p-value** |
| --- | --- | --- | --- | --- | --- |
| *Wrist Velocity* | first half | 705.965 ± 24.246 |  |  |  |
|  | second half | 657.52 ± 20.757 | 5.521 | 1, 260.37 | 0.027* |
| *Wrist Acceleration* | first half | 14249.848 ± 412.432 |  |  |  |
|  | second half | 13185.936 ± 330.139 | 8.688 | 1, 260.20 | 0.012* |
| *Grip Aperture* | first half | 127.811 ± 2.265 |  |  |  |
|  | second half | 129.475 ± 2.072 | 0.628 | 1, 260.07 | 0.429 |
| *Wrist Height* | first half | 43.824 ± 1.666 |  |  |  |
|  | second half | 45.023 ± 1.361 | 6.037 | 1, 261.03 | 0.027* |

(*p < 0.05; **p < 0.01, ***p < 0.001)
